# Buccal Bullfrog (*Rana catesbeiana* Shaw) Oil Emulsion: A Mucoadhesive System Intended for Treatment of Oral Candidiasis

**DOI:** 10.3390/pharmaceutics10040257

**Published:** 2018-12-03

**Authors:** Susiane S. Moreira-Oliveira, Lucas Amaral-Machado, Wógenes Nunes de Oliveira, Éverton N. Alencar, Kelly Cristine Zatta, Luanda B. F. C. de Souza, Aldo da Cunha Medeiros, Guilherme Maranhão Chaves, Eryvaldo S. T. Egito

**Affiliations:** 1 Graduate Program in Health Sciences, Federal University of Rio Grande do Norte (UFRN), Av. General Gustavo Cordeiro de Faria-SN-Petrópolis, Natal 59012-570, Brazil; susiane.m@gmail.com (S.S.M.-O.); machado.lucasam@gmail.com (L.A.-M.); guilherme.chaves@ufrnet.br (G.M.C.); 2 Graduate Program in Pharmaceutical Sciences, UFRN, Av. General Gustavo Cordeiro de Faria-SN-Petrópolis, Natal 59012-570, Brazil; wogenes95@gmail.com (W.N.d.O.); luanda_canario@hotmail.com (L.B.F.C.d.S.); 3 Laboratory of Dispersed Systems (LaSiD), UFRN, Av. General Gustavo Cordeiro de Faria-SN-Petrópolis, Natal 59012-570, Brazil; everton_alencar@hotmail.com; 4 Faculty of Pharmacy, Federal University of Rio Grande do Sul (UFRGS), Av. Ipiranga-2752-Santana, Porto Alegre 90610-000, Brazil; kellycriz@hotmail.com; 5 Department of Surgery, UFRN Av. General Gustavo Cordeiro de Faria-SN-Petrópolis, Natal 59012-570, Brazil; aldom@uol.com.br; 6 Laboratório de Sistemas Dispersos, Departamento de Farmácia, Universidade Federal do Rio Grande do Norte, Rua Praia de Areia Branca, 8948, Natal 59094-450, RN, Brazil

**Keywords:** bullfrog oil, buccal emulsion, mucoadhesivity, antifungal activity, biocompatibility

## Abstract

Oral candidiasis (OC) is an infectious disease caused by microorganisms of the genus *Candida*, leading to lesions in the buccal cavity. Its treatment consists of the administration of topical or systemic antifungal agents, which may compromise the patient compliance due to its side effects, highlighting the need for alternative treatments. In this scenario, bullfrog oil, an animal oil composed of a pool of saturated and unsaturated fatty acids, is introduced as a potential antifungal raw material. Thus, the aim of this work was to produce a mucoadhesive emulsified system able to deliver the bullfrog oil in the buccal cavity to treat the OC. The emulsion was produced and characterized by visual inspection, droplet size, polydispersity index (PdI), and zeta potential over the course of 60 days. In addition, its mucoadhesive ability was evaluated using an in vitro mucin model. The antifungal activity, evaluated by the broth microdilution assay and the biocompatibility, performed against human erythrocytes, were also carried out. The emulsion showed a droplet size of 320.79 ± 35.60 nm, a PdI of 0.49 ± 0.08, and a zeta potential of −38.53 ± 6.23 mV, with no significant changes over 60 days. The mucoadhesive properties of the system was improved by the use of pharmaceutical excipients. The antifungal activity showed that the bullfrog oil and the emulsion were able to inhibit the growth of different *Candida* species. Furthermore, the emulsion showed no significant hemolytic effect. Overall, the system showed suitable physicochemical characteristics and biocompatibility, with substantial in vitro antifungal activity, suggesting that this system can be further investigated for OC treatment.

## 1. Introduction

Oral infections are a group of widespread diseases that affect individuals of all ages and socioeconomic classes, often triggered by commensal microorganisms, which may become pathogenic due to inappropriate buccal hygiene or trauma, mainly when the patient’s immunity is compromised [1]. In addition, the oral cavity offers excellent conditions for microorganism growth, such as adequate pH and temperature, high humidity, substrates and hydrolytic enzymes, such as proteases, phospholipases, and haemolysin, able to promote food cleavage, delivering free glucose to the microorganism and thus promoting the quick development of these infections [2,3,4].

Among oral infections, oral candidiasis (OC) represents the most frequent oral fungal infection, caused by the photogenic growth of different *Candida* species, such as *Candida albicans*, *Candida glabrata*, *Candida parapsilosis*, and *Candida tropicalis*, which are responsible for 30 to 45% of oral fungal infections in the general adult population [5,6,7]. Risk factors for oral candidiasis include use of dentures, xerostomia, prolonged therapy with antibiotics, local trauma, malnutrition, and endocrine disorders [8]. Oral candidiasis is one of the most common clinical symptoms of patients infected with the human immunodeficiency virus (HIV) and transplanted patients [4,7,8]. The pathogenicity of these species is assigned to virulence factors that promote the adhesion to host cells, affecting the buccal keratinized mucosa and oral prosthesis, with possible biofilm formation on host tissues or medical devices, contributing to the maintenance of the infection, allowing the microorganism to escape from host defense mechanisms [9] and increasing its ability to invade the colonized tissue, promoting oral mucosa damage [9,10,11].

Therefore, OC can be classified according to its clinical aspects such as the presence or absence of desquamated epithelial cells, angular cheilitis, gingival erythema, and presence of necrotic material [6,12]. Thus, OC presents two classifications, (i) pseudomembranous OC, which is characterized by white lesions, foul breath, and hyperplasic candidiasis and (ii) erythematous OC, which presents red lesions, and acute or chronic atrophic candidiasis [12].

Based on the clinical analysis and the microorganism characteristics, OC treatment consists of the elimination of the identified predisposition factors through buccal and prosthesis hygiene as well as the use of topical or systemic antifungal agents [13,14]. The regular teeth, buccal cavity, and denture cleaning with antiseptic rinses containing chlorhexidine or hexetidine is an effective approach to reduce *Candida* spp. load in the oral cavity [5,8]. However, the use of topical or systemic antifungal agents such as nystatin, miconazole, clotrimazole, fluconaloze, itraconazole, echinocandins, flucystein, and amphotericin B represent other important treatment strategies for OC, especially when the patient’s immunity is a relevant aspect to be considered [5,6,12].

These therapeutic strategies are effective to treat oral *Candida* spp. infections since they are able to promote the decrease or complete removal of pathogenic fungal strains in the oral cavity. Nonetheless, these drug treatments are also responsible for promoting several side effects, such as teeth and tongue discoloration (chlorhexidine), nephrotoxicity, hepatotoxicity, polyuria, skin rashes, acne, nausea, chest pain, and gastrointestinal disturbance (topical and systemic antifungal agents), compromising the treatment compliance and the patient’s life quality [5,12,13].

Thus, based on the high prevalence of *Candida* spp. infections in the oral cavity and the side effects related to its treatment, it has become evident the need for new alternative treatments to overcome this problem, especially for non-extensive lesions of OC, since that these new alternatives will make it possible to treat the infection without exposing the patient to the side effects of traditional therapies [15]. In this context, studies that evaluated the antifungal activity of natural products, such as plant extracts and natural oils, highlighted that these products are able to inhibit the growth of *Candida* spp. [5,15], suggesting potential use of natural products in OC treatment.

Among these products, bullfrog oil stands out as an animal oil extracted by the biotechnological reuse of the adipose tissue from the amphibious *Rana catesbeiana* Shaw. Bullfrog oil presents in its chemical composition a pool of saturated and unsaturated fatty acids responsible for potential antimicrobial and antibiofilm activities [16,17]. Nevertheless, the in natura use of this oil shows some disadvantages, such as unpleasant organoleptic characteristics and undesirable biopharmaceutical properties, which may compromise its use and leads to a low patient compliance [16,17]. To overcome these drawbacks, the development of an emulsified system to deliver the bullfrog oil and to allow its adherence to the buccal mucosa could be a technological approach that enables its use for treatment of OC. Examples of emulsified systems are microemulsion [18], topical nanoemulsion for external use [17], and oral nanoemulsion [19], already developed by our research group. Indeed, such systems, composed of aqueous and oily phases stabilized by a surfactant blend [20], allow the internalization of bullfrog oil in its droplets and, through the use of pharmaceutical excipients, may increase the adherence of this oil into the buccal mucosa, allowing contact to the microorganisms of the oral cavity, and consequently improving its antimicrobial activity [21].

Thus, the aim of this study was to produce an emulsified system for buccal administration based on bullfrog oil and to evaluate its stability, in vitro biocompatibility, mucoadhesive properties and antimicrobial activity in microorganisms responsible for promoting OC.

## 2. Materials and Methods

### 2.1. Materials

#### 2.1.1. Chemicals

Bullfrog oil was provided by Asmarana Produtos Naturais (Natal, Brazil). Sodium hydroxide (NaOH), dimethyl sulfoxide (DMSO), potassium hydroxide, diethyl ether, hydrochloric acid (HCl), Tween^®^ 20, sodium thiosulfate, and sodium carbonate were from VETEC (Rio de Janeiro, Brazil). Miglyol^®^ 812 was a gift from Sasol (Witen, Germany). Butylhyldroxytoluene (BHT), buthylhydroxyanisole, potassium biphthalate, potassium dichromate, and Wijs solution were purchased from Labsynth (São Paulo, Brazil). Acetic acid, ethanol P.A, sodium bicarbonate, potassium iodide, starch, and chloroform were from Isofar (Rio de Janeiro, Brazil). Sucralose, Tutti-frutti flavoring and Acesulfame K were purchased from Valdequímica (São Paulo, Brazil). Xanthan gum, sodium benzoate and propylparaben were from ViaFarma (São Paulo, Brazil). Phenolphthalein was provided from Biotec Chemicals (Londrina, Brazil). Mucin Type II (Mucin from porcine stomach) and Span^®^ 80 were from Sigma-Aldrich (São Paulo, Brazil).

#### 2.1.2. Biological

*Candida* spp. strains used during the experiments were *Candida albicans* ATCC 90029, *Candida dubliniensis* CBS 7987, Candida glabrata ATCC 2001, *Candida parapsilosis* ATCC 22019, *Candida metapsilosis* ATCC 96143, Candida orthopsilosis ATCC 96139, and *Candida tropicalis* ATCC 13803, donated by the culture collection of the Laboratory of Medical and Molecular Mycology from the Federal University of Rio Grande do Norte (UFRN) (Natal, Brazil). The blood sample was kindly donated by the Hemocenter Dalton Cunha (Natal, Brazil).

### 2.2. Methods

#### 2.2.1. Physicochemical Characterization of Bullfrog Oil

Physicochemical analyses were performed to evaluate the quality of the bullfrog oil according to the adapted titration methods described in the United States Pharmacopeia (USP 35) [22] and the American Oil Chemists Society guidelines [23] for peroxide index (PI), acid index (AI), iodine index (II), and saponification index (SI). The PI was determined using 0.5 g of bullfrog oil dissolved in 3 mL of acetic acid: chloroform solution (3:2 *v*/*v*) and 0.1 mL of saturated solution of potassium iodide. After complete dissolution, 3 mL of purified water was added and the mixture was titrated with 0.01 N sodium thiosulfate solution using starch as indicator. The AI was determined by the titration of 0.5 g of bullfrog oil solubilized in 2.5 mL ether-alcohol solution (1:1 *v*/*v*) with sodium hydroxide (0.1 N) using phenolphthalein as indicator. In addition, the II was determined by the titration of 0.2 g of bullfrog oil, 8 mL of chloroform, and 20 mL of wijs solution. This mixture was kept under dark conditions for 30 min and, then, 30 mL of potassium iodide 15% and 80 mL of water were added for titration with sodium thiosulfate (0.1 N) using starch as indicator. The SI was assessed using 1 g of bullfrog oil and 25 mL of alcoholic solution of potassium hydroxide (0.5 N). This mixture was titrated with hydrochloric acid (0.5 N) using alcoholic phenolphthalein 1% as indicator.

#### 2.2.2. Production of Buccal Emulsified System Containing Bullfrog Oil

The buccal emulsion based on bullfrog oil (BBE) (Table 1) was prepared by the phase inversion technique [24]. Its composition was based on a previous study performed by our research group [19]. First, the aqueous and oily phases were heated separately at 70 °C and, then, the aqueous phase was transferred to the oily phase under constant stirring at 11,000 rpm for 10 min using an Ultra-Turrax^®^ T-18 (IKA, Staufen, Germany).

In addition, a blank emulsion (BE) and a buccal Mygliol^®^ 812 (a medium chain triglyceride mixture of capric and caprilic fatty acids) emulsion (BME), based on the same method, were also produced for use in the in vitro mucoadhesive and the antifungal/biocompatibility studies, respectively. The BE formulation was composed by bullfrog oil (14%), distilled water (80%) and surfactant blend (Tween^®^ 20 and Span^®^ 80 at proportion of 1.72:1 *w*/*w*) (6%). On the other hand, the BME and the BBE have similar composition (Table 1).

#### 2.2.3. Buccal Bullfrog Oil Characterization

The emulsions characterization studies were performed 24 h after their production and the system stability was evaluated over the period of 60 days.

#### 2.2.4. Macroscopic Aspects

Organoleptic characteristics (color and odor) and macroscopic aspect of the developed system were analyzed by visual inspection in order to verify the presence of sensorial changes and macroscopic instability phenomena such as creaming or phase separation. Test conditions included storage in a translucent test tube and temperature of 25 ± 2 °C.

#### 2.2.5. pH and Conductivity Evaluation

The pH and the electrical conductivity were analyzed at 25 ± 2 °C, in triplicate, using a Tecnal pH-meter model TEC-2 (Piracicaba, Brazil) and a Digimed conductivimeter model DM-32 (São Paulo, Brazil).

#### 2.2.6. Droplet Size Distribution and Zeta Potential Analysis

The measurement of the droplet size distribution was performed in triplicate by dynamic light scattering (DLS) technique using a ZetaSize NanoZS (Malvern Instruments, Malvern, UK) at 25 °C and angle fixed at 173 °C. The samples were previously diluted (1:200 *v*/*v*) in purified water according to the dilution study (data not shown). Additionally, the zeta potential was analyzed by eletrophoretic mobility using the ZetaSize NanoZS at 25 °C. In order to maintain the ionic strength, the samples were diluted (1:200 *v*/*v*) in sodium chloride solution at 0.1 mM and the measurement was performed in triplicate.

#### 2.2.7. Viscosity Measurements

The viscosity evaluation of BBE was performed in triplicate at 25 ± 2 °C using a cone and plate (CP) rheometer (Brookfield-model RV-III, New Castle, DE, USA) equipped with a CP 42 spindle. The sample (1 g) was placed in the spindle and the viscosity was measured under progressive rotation between 35–60 rpm with interval of 5 rpm. The rotation was maintained for 10 s at each speed and the data of shear rate, shear stress and viscosity were analyzed by the Rheocalc V 3.01 software.

#### 2.2.8. In Vitro Mucoadhesive Studies

##### Mucoadhesive Performance

The mucoadhesive properties of the BBE and the BE were assayed by a mucoadhesive test method [25] using a texture analyzer (TA.XT Plus Texture Analyzer, Hamilton, MA, USA).

Initially, mucin disks were prepared as a model membrane using a manual tablet press machine, Korsh, Model EK-0 (KORSH America Inc., South Easton, MA, USA) (12 mm diameter punch, 172 ± 2 mg), which were fixed at the superior probe of the texturometer and washed with 200 µL of ultrapure water at 37 °C for 2 min. The BBE and the BE were added to the texturometer inferior probe and maintained at 37 °C during the test execution. Posteriorly, the superior probe (initial height of 700 mm) was moved down at the speed of 2 mm·s^−1^ until contact with the emulsions by applying a minimum force (0.2 N–0.05 mm·s^−1^; during 300 s). The probe returned to the initial stage at the same speed and the mucoadhesion work (*W*_ma_) was measured according to the force required to separate the two probes and the distance of stretching to detach the samples from the mucin.

##### Interaction between the Emulsion and the Mucin

The ability of the BBE and the BE to interact with the mucin was evaluated by means of the droplet size and the zeta potential, which were measured before and after contact with mucin solutions at different concentrations (200, 250, and 350 µg·mL^−1^) [26].

Initially, the droplet size and the zeta potential were evaluated using a ZetaSize NanoZS (Malvern Instruments, Malvern, UK). Then, 50 µL of the emulsions were added in 10 mL of the mucin solutions and kept for 1 h at 37 °C under moderate magnetic stirring. Subsequently, the droplet size and the zeta potential were reevaluated under the same dilution condition as described in Section 2.2.6. All analyses were performed in triplicate. 

#### 2.2.9. Antimicrobial Activity of Bullfrog Oil and Buccal Bullfrog Oil Emulsion

##### Inocula Preparation

Fungal strains were previously reactivated on sabouraud-dextrose agar (SDA) for 48 h. Subsequently, yeast cells inocula were standardized in tubes containing 5 mL of sterile saline solution at 0.9% and the microbial suspension was adjusted to the 0.5 McFarland standard by absorbance measurement using an UV-Vis spectrophotometer (Biochrom^®^, Libra^®^ S32, Cambourne, UK).

##### Fungal Minimal Inhibitory Concentration (MIC)—Broth Microdilution Assay

The fungal MIC was performed using the biological strains aforementioned (Section 2.1.2). The inocula were diluted in Mueller–Hinton broth (MHB) at the ratio of 1:30 according to an adaptation from the method described by Oliveira et al. (2018) [18]. The BBE and the BME were diluted in sterile MHB and bullfrog oil was diluted with DMSO at 1% in sterile MHB in order to obtain an oil concentration of 4 mg·mL^−1^ for all samples. The DMSO solution at 1% and the BME were used as negative control. Serial dilutions (emulsion: MHC, 1:2 *v*/*v*) were prepared with sterile MHC in a 96-well microplate and incubated at 37 °C ± 2 °C for 48 h. The fungal MIC was considered as the lowest concentration of the samples able to inhibit 100% growth of each strain by visual inspection, according to the recommended by the Clinical Laboratory Standards Institute [27].

#### 2.2.10. In vitro Biocompatibility Assay—Hemolysis in Total Blood

In order to evaluate the hemolytic potential of the bullfrog oil, the BBE, and the BME, an O^+^ blood sample from a healthy donor was used aiming to evaluate the biocompatibility. The hemolytic potential of the Miglyol^®^ 812 used in the BME was also assessed. Thus, the blood sample was centrifuged at 1100 g for 10 min and the plasma was removed. Posteriorly, the erythrocytes suspension was washed three times with 0.9% saline solution and the cells concentration adjusted to 5% (*v*/*v*) (6 × 10^6^ cell/mL) in 0.9% of saline solution.

The oils, bullfrog oil and the Miglyol^®^ 812, were diluted in DMSO at 1% saline solution and the emulsions, the BBE and the BME, were diluted in saline solution in order to reach oil concentration of 1.0 mg·mL^−1^, 0.50 mg·mL^−1^, and 0.25 mg·mL^−1^. Thus, 1.5 mL of erythrocytes suspension were incubated with 1.5 mL of bullfrog oil, BBE, and BME at the concentrations described above for 1 h at 37 °C. After incubation, the samples were centrifuged at 1100 g for 5 min and the supernatant was removed and directly read in a spectrophotometer (Biochrom^®^, Libra^®^ S32, Cambourne, UK) set at 540 nm. The analysis was performed in triplicate. Turk’s solution at 1% and saline solution at 0.9% were used as positive and negative control, respectively. Finally, the hemolytic potential was calculated according to the Equation (1) [18]:

(1) $$Hp\left(\%\right)=100\times \frac{As-An}{Ap}$$

where, *Hp* = Hemolytic potential (in percentage); *As* = Absorbance of tested sample; *An* = Absorbance of the Negative Control; *Ap* = Absorbance of the Positive Control.

### 2.3. Statistical Analyses

The results were expressed as mean ± standard deviation. Student’s *t*-test was used between two unpaired groups and *p* values less than 0.05 were considered significant. In addition, statistical significance among groups was evaluated by Analysis of Variance analysis (ANOVA) followed by Tukey’s post-test.

## 3. Results

### 3.1. Bullfrog Oil (Rana catesbeiana Shaw) Physicochemical Characterization

In order to assess the physicochemical properties of bullfrog oil, as well as the quality parameters related to its use as pharmaceutical raw material, the peroxide index (PI), the acid index (AI), the iodine index (II), and the saponification index (SI) were evaluated through titration techniques.

The PI is a parameter widely used to measure the lipid hydroperoxides, a primary product of the lipid oxidation. This process affects the organoleptic characteristics (odor and appearance) and quality of natural oils, as it might lead to the formation of toxic compounds due to the decomposition of unsaturated and polyunsaturated fatty acids presented in the natural oils [28,29]. The bullfrog oil *PI* value was 1.90 ± 0.03 mEq O_2_/Kg. Similar data was found by Rutckeviski and colleagues (2017) for a bullfrog oil sample obtained by hot extraction at 70 °C, which showed *PI* value of 1.93 ± 0.02 mEq O_2_/Kg [30]. Our result indicates that this marketable bullfrog oil has suitable quality, with low oxidation levels, evidencing a preservation of the unsaturated and polyunsaturated fatty acids, as shown for the bullfrog oil obtained by the study that evaluated different extraction processes [30].

Furthermore, the AI was also evaluated since this parameter measures the free fatty acid content and indicates the hydrolytic degradation process caused by the presence of water molecules and lipases enzymes which promotes fatty acid cleavage, compromising the oil quality [31]. The bullfrog oil showed an AI value of 1.57 ± 0.01 mg KOH/g oil, which is in agreement to the Codex Alimetarius for Edible Oils (*AI* ≤ 3 mg KOH/g oil) [32], suggesting a lower content of free fatty acids and suitable conservation of the bullfrog oil. This result is in agreement with the literature, where an AI value near 2.90 mg KOH/g oil was obtained in the bullfrog oil extracted from the adipose tissues of the amphibious *Rana catesbeiana* Shaw by hot and organic solvent extraction processes [17].

Additionally, the II and the SI were assayed to evaluate both the unsaturation degree of the fatty acids presented in the oil composition and the chain length of the fatty acids, which allow us to predict the purity and the quality of the oil, respectively [17,30]. The bullfrog oil showed an *II* value of 99.60 ± 1.24 g I_2_/100 g oil and SI of 169.52 ± 0.83 mg OH/g oil. These data were in accordance with the literature, where the *II* value was 104.21 ± 1.00 g I_2_/100 g and the *SI* value was 171.12 ± 0.97 mg OH/g oil for bullfrog oil extracted through a hot process at 70 °C [30] and supported by the chemical composition of the bullfrog oil, as already described [17].

Overall, the results of the physicochemical evaluation allow us to suggest that the bullfrog oil used in this work shows valuable characteristics, such as a low PI and AI, and II and SI similar to the literature reports, indicative of the absence of degradative phenomena that may compromise the oil quality, allowing the use of bullfrog oil as a potential raw material on the development and production of pharmaceutical products.

### 3.2. Production of Buccal Bullfrog Oil Emulsion (BBE)

The results from the physicochemical analyses revealed that the bullfrog oil presented suitable characteristics to be used on the buccal emulsion. An emulsified system was chosen due to its ability to improve the biopharmaceutical properties and the organoleptic characteristics of drugs and oils, respectively. In addition, these systems have also the ability to enhance the drugs and the bioactive therapeutic effects, reducing doses, side effects, and toxicity, allowing the improvement of the treatment, their efficacy, and patient compliance [21].

On the other hand, due to the large interfacial area presented in the emulsified systems, the occurrence of instability phenomena such as flocculation, coalescence, creaming, and phase separation over the time was accentuated, demonstrating the importance of the use of pharmaceutical excipients to improve emulsion stability [33,34]. Thus, an experimental design study was previously performed to define the best pharmaceutical excipients and their concentrations, improving the organoleptic characteristics of the bullfrog oil and thus enabling the system to be used by oral and buccal routes [19].

The BBE showed a milky and homogeneous appearance with the characteristic smell of the tutti-frutti flavor used in this formulation, which masked the undesirable smell of bullfrog oil. These macroscopic aspects allow us to suggest that this system was able to improve the organoleptic characteristics of bullfrog oil.

Additionally, the physicochemical aspects (pH, conductivity, droplet size, polydispersity index, and zeta potential) were also assessed. The emulsion pH value was slightly acidic (6.05 ± 0.02) which was maintained over time, showing a non-significant decrease at the end of 60 days (5.76 ± 0.17). This slightly acid characteristic may be attributed to both the fatty acids presented in the chemical composition of the bullfrog oil [18]. Similar data were reported by the literature in which distinct emulsified systems based on bullfrog oil such as nanoemulsion and microemulsion showed pH values of 6.3 ± 0.2 and 6.1 ± 0.1, respectively [17,18].

Hence, the produced BBE presents itself as a compatible system able to be administrated to the buccal mucosa, avoiding discomfort during the administration, since that the buccal mucosa has a pH range between 5.5 and 7.0, fluctuating according to the salivation flow rate [35]. In addition, it is possible that the use of preservatives and antioxidants in the emulsion prevented the microbial contamination and chemical degradation of emulsion components, which could lead to considerable pH decrease [17].

Furthermore, high conductivity values reinforced the characterization of the formulation as an O/W system, since this parameter indicates the external phase characteristic of the emulsion [21]. The BBE showed a high conductivity value (1932.00 ± 10.15 µS·cm^−1^), which indicates an external aqueous phase, similar to the water conductivity and characteristic of the oil-in-water (O/W) emulsions [21]. In addition, this data did not change significantly throughout the stability study (1848.33 ± 42.72 µS·cm^−1^), indicating no phase inversion, phase separation, or degradation of oil droplets.

Moreover, the stability of the BBE was also followed by the analysis of the droplet size and the zeta potential, which are parameters related to the physical and electrostatic stability of emulsified systems, respectively [21,36]. Indeed, the monitoring of the droplet size allows the identification of instability phenomena that are not detected by visual inspection, such as flocculation (aggregation/approximation of the oil droplets due the interaction energy and low distance between the droplets) and coalescence (collision/fusion of two or more oil droplets to form a single larger droplet) [36,37].

The BBE showed a droplet size of 320.79 ± 35.60 nm and a polydispersity index (PdI) of 0.49 ± 0.08. Similar results for droplet size of nanoemulsions can be found in the literature [21,36]. Droplet size data also contributes to the classification of emulsified systems as macroemulsions (>1 µm), nanoemulsions (size around 300 nm), and microemulsions (size lower than 100 nm). However, it is important to highlight that the evaluation of other parameters such as visual appearance, polydispersity index, and kinetic and thermodynamic stability, needs to be also considered for a correct classification [21,36]. For instance, nanoemulsions show low PdI, kinetic stability, and particular visual characteristics (Tyndall effect) that are not observed in the BBE [21,38]. Thus, according to the literature, the BBE can be classified as a macroemulsion, since it presented droplet size in the range of 100 nm^–1^ µm, elevated PdI, and no Tyndall effect [38].

**Figure 1:**
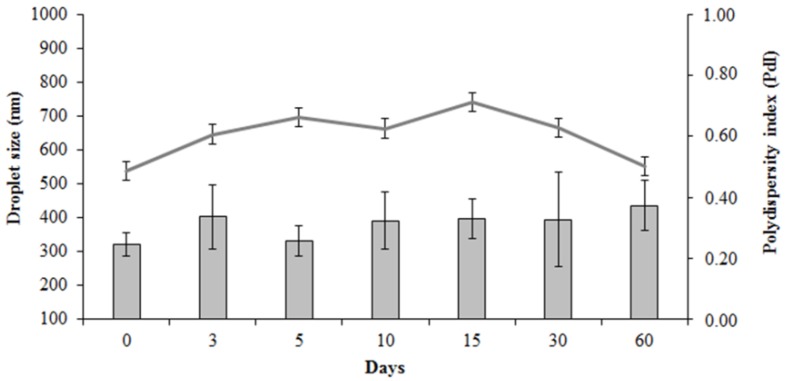
Droplet size (column graph) and polydispersity index (PdI) (line graph) of the buccal bullfrog oil emulsion stored at 25 ± 2 °C for 60 days.

Additionally, at the end of the stability study (Figure 1) an increase in the droplet size was observed. Right after its production, the droplet size was 320.79 ± 35.60 nm, whereas after 60 days an increase to 436.10 ± 72.70 nm was observed. The PdI remained constant (from 0.49 ± 0.08 to 0.50 ± 0.02) throughout the study, suggesting an absence of instability phenomena that may lead to phase separation [37,38]. Based on these results, it is possible to suggest that BBE may be used on the treatment of no extensive lesions of OC. In fact, *Candida* species exhibits cells with oval to round shape with a diameter of 3.5–8 µm [39] and the BBE with the aforementioned droplet size may be adsorbed at the *Candida* spp. wall, promoting the bullfrog oil effect directly over the fungal cell.

The zeta potential of the BBE was also evaluated over 60 days. This parameter is directly related to the electrostatic stability of emulsified systems because the electrical charge promotes droplet repulsion, avoiding flocculation [37]. Thus, absolute values of zeta potential higher than 30 mV indicate that the repulsive forces are stronger than the attractive ones, impeaching the oil droplets approximation and further instability [37,40]. The BBE showed an initial zeta potential value of −38.53 ± 6.23 mV, suggesting electrostatic stability, corroborating to the data observed for a topical nanoemulsion produced with bullfrog oil in its internal phase, as described in the literature [17]. In addition, over the period of 60 days, the zeta potential of the BBE remained steady (−33.77 ± 4.03 mV), data that allows us to conclude that the emulsion presents a suitable electrostatic behavior regarding its ability to avoid instability phenomena that may compromise its quality. Furthermore, the BBE viscosity was also evaluated according to the shear rate and the shear stress variations. This analysis suggested the rheological behavior of the system, predicting its spreadability and residence time in the biological surface of the buccal mucosa [41,42]. Furthermore, a non-Newtonian pseudoplastic behavior was observed with the shear rate and the shear stress variations, since the viscosity decreased according to the increase of these parameters (data not shown). For a shear rate of 228 s^−1^ at 25 ± 2 °C, the BBE showed a viscosity of 39.0 ± 0.2 cP. These results indicate a suitable viscosity and rheological characteristic of BBE, once the pseudoplastic behavior is desired to the buccal delivery, allowing the easy spreadability of the system, and thus, improving its contact with the buccal mucosa cell [42].

Therefore, the overall results suggest that the BBE has suitable physicochemical characteristics, which remains stable for 60 days, and suitable viscosity, which predict good rheological behavior for buccal delivery.

### 3.3. Evaluation of Mucoadhesive Properties of the Buccal Bullfrog Oil Emulsion

The epithelium surface of the buccal mucosa is covered by a mucus layer responsible for its lubrication, protection, and hydration. This layer is composed by a viscoelastic network of water (95%) and mucin (5%), a glycoprotein responsible for the viscosity and gel properties of the mucus [26,43]. Thus, many studies have evaluated the interaction between dispersed systems and this glycoprotein with the purpose of predicting the mucoadhesion ability of these systems, which is related to increased interactions with the buccal mucosa and site-specific delivery of drugs and/or bioactive compounds [26,44]. Based on this rationale, the BBE was designed to be used on the buccal mucosa as potential treatment to non-extensive lesions of OC. Its ability to adhere to the buccal epithelium was assessed by in vitro mucoadhesion measurements using two different approaches: (i) the mucoadhesion performance and (ii) the variation in droplet size and zeta potential due to the interaction between the emulsion and the mucin [25,26].

The mucoadhesion performance was carried out by the tensile stress test, which consists in a specific apparatus in which the emulsions were placed into an inferior probe that interacts with a superior probe, containing a previously prepared mucin membrane, for a specific time. After the interaction, the maximum force and work required to separate the probes were calculated considering the debonding distance, thus reflecting the mucoadhesion properties of the analyzed system [25,42]. Table 2 shows the parameters related to the mucoadhesion performance of the BBE and the BE, which was produced without pharmaceutical excipients.

The BE showed a maximum peak force of 7.44 ± 1.53 mN to be detached from the mucin membrane, with a debonding distance of 949.94 ± 92.72 mm, resulting in *W_ma_* of 1080.96 ± 204.68 mN·mm, suggesting a low adhesion behavior. On the other hand, the BBE required a significantly higher force to detach from the mucin membrane and showed peak force of 10.15 ± 2.00 mN with a longest debonding distance of 1752.36 ± 215.53 mm, resulting in a higher *W_ma_* (1494.04 ± 203.45 mN·mm) compared to the BE (*p* < 0.05), highlighting the superior capacity of the BBE to adhere to the mucin membrane. This data can be attributed to the composition of the BBE if it presents pharmaceutical excipients, such as xanthan gum and sweeteners, which may improve the adhesion ability of the system. In fact, the xanthan gum is a anionic polymer widely used in pharmaceutical systems due to its mucoadhesive properties, since its molecule presents a helical form with D-glucose residues and glucuronic acid groups between mannose units, providing a steric conformation able to form secondary bonds with the human mucin [45,46]. The mucin type II used in this test is derived from the porcine stomach and is chemically and structurally similar to human mucin, allowing us to suggest that BBE is able to interact with the mucus from the human buccal mucosa and to deliver the bullfrog oil [47,48].

Additionally, the mucoadhesive potential of the BBE and the BE was also assayed by the measurement of the droplet size and the zeta potential of these systems before and after contact with mucin solutions at different concentrations, as described in the Table 3. Initially, the BBE showed a droplet size of 320.79 ± 35.60 nm and a zeta potential value of −38.53 ± 6.23 mV, whereas the BE presented a droplet size of 186.7 ± 2.7 nm and a zeta potential of −18.20 ± 1.42 mV. As seen in Table 3, both emulsions had a significant increase in droplet size, suggesting the interaction of the mucin molecules with the oil droplet surface. In addition, a significant statistical difference was observed in all tested concentrations (*p* < 0.05) on the increase of droplet size between the BBE and BE, which implicates that the droplet size of the BE is higher than that of the BBE after mucin contact. In fact, the interaction between the mucin molecules and the oil droplets is a phenomenon described in the literature [49,50]. Due to the ability of mucin to promote chemical interactions with the surface of oil droplets, large aggregates are formed due to the occurrence of flocculation involving the oil droplets, resulting in a significant increase in droplet size. Furthermore, as the BE did not have stabilizing agents in its composition, the mobility and the contact of the oil droplets with the mucin molecules was facilitated, allowing their interaction, and resulting in a noticeable increase in the droplet size.

On the other hand, due to the presence of pharmaceutical excipients in the external phase of the BBE, the mucin molecules presented in the dispersion may have a difficulty on interacting with the oil droplets, once some excipients, such as the xanthan gum, are able to chemically interact with the mucin molecules as previously discussed. Thus, these data allow us to suggest that the non-expressive increase in the droplet size of BBE, when compared to the BE, can be attributed to the reduced amount of free mucin available to interact with the oil droplets, since these molecules are possibly interacting with the xanthan gum in the external phase.

Additionally, Table 3 also shows the zeta potential of the BBE and the BE. It is possible to observe a difference in this parameter for both systems, reinforcing the hypothesis that mucin was able to adhere to the oil droplets surface. Indeed, this molecule presents sialic acid residues linked to the terminal region of the oligosaccharide chains that lead to a negative surface charge [25,26], which makes this molecule able to adsorb on the particles and/or the oil droplets, promoting changes in the physicochemical characteristics of the system, as described on the literature [26,49,51]. Furthermore, although both samples and mucin solutions have a negative charge, their interaction is possible because the negative charge of the mucin solutions was close to the isoelectric point, allowing the electrical and chemical interaction of the oil droplets and preventing the electric repulsion. In addition, it was also observed that the zeta potential of the BBE increased according to the mucin concentration in the dispersions, data not observed for the BE, corroborating to the results obtained by the tensile stress test and highlighting the superior mucoadhesive ability of the BBE.

Additionally, it is possible to compare the results obtained by these two different approaches used to assess the mucoadhesivity. Indeed, the mucoadhesion study, performed by the tensile stress, showed an increase of the work forces necessary to separate the probes after contact to the BBE compared to the BE. On the other hand, the variation of the droplet size and the zeta potential showed higher interaction between the mucin molecules to the BE droplets compared to the BBE, which allowed mucin to be available on the external phase and cause mucoadhesion. Based on the fundamentals of each performed test, these results corroborate to the hypothesis that the pharmaceutical excipients were able to increase the physical interaction between the system and the mucosa.

Thus, these results allow us to suggest that the pharmaceutical excipients used in the production of the BBE, such as the xanthan gum, were able to increase the interaction forces impacting in the mucoadhesion ability of this system, which may increase its contact time with the buccal mucosa, leading to site-specific delivery of the bullfrog oil.

### 3.4. Antifungal Activity of Buccal Bullfrog Oil Emulsion

In order to evaluate the antifungal activity of the bullfrog oil and the BBE, a broth microdilution assay was performed. This method is an economic and effective approach that allows the determination of the lowest concentration of the tested agents able to inhibit the growth of different yeasts strains completely [52]. The DMSO 1% and the BME were used as negative control and did not show antifungal activity against the tested *Candida* spp.

The pure bullfrog oil was able to inhibit the growth of distinct *Candida* spp. reference strains, except *C. dubliniensis* CBS 7987 and *C. glabrata* ATCC 2001, with MIC values in the range of 0.25–0.5 mg·mL^−1^, while the BBE showed antifungal activity against all the *Candida* spp. strains tested with a MIC ranging from 0.5 to 1.0 mg·mL^−1^ (Table 4). The absence of antifungal effect of the pure bullfrog oil against the *C. dubliniensis* and *C. glabrata* reference strains can be related to the resistance profile of these yeast strains, or also due to the fungal cell structure (fungal cell wall composition or the fungal phospholipidic membrane) [53]. This hypothesis can be reinforced by the literature, which describes that due to the fungal cell structure, different *Candida* spp. can present variations in the wall composition and organization, reflecting directly on the antifungal activity of natural oils in its natural form [16,54]. On the other hand, the BBE showed MIC values of 0.5 mg·mL^−1^ and 1.0 mg·mL^−1^ against *C. dubliniensis* CBS 7987 and *C. glabrata* ATCC 2001, respectively. This result can be attributed to an improvement of the absorption of natural oils by the fungal cells caused by emulsified systems, since the emulsion composition can interact with the fungal cell wall or even with the phospholipidic structure of the fungal cell membrane, promoting the interaction between the oil and the microorganism and thus improving its antifungal activity [16,21].

Additionally, it was possible to observe that the bullfrog oil showed MIC values ranging from 0.25 mg·mL^−1^ to 0.50 mg·mL^−1^ against other yeast strains, while the BBE presented MIC values from 0.50 mg·mL^−1^ to 1.0 mg·mL^−1^. These results highlight the antifungal activity of both bullfrog oil and the produced emulsion against different *Candida* species responsible for causing OC [12,55]. When compared to other natural oils used in the Brazilian medicine against *C. albicans* infections, bullfrog oil and the BBE were more potent than *Anthemis nobilis* L. (MIC 0.8 mg·mL^−1^), *Baccharis trimera* DC. (MIC 2.0 mg·mL^−1^), and *Mentha pulegium* L. (MIC 0.74 mg·mL^−1^) oils [56].

It’s important to highlight that the MIC values of the BBE were higher than that of the pure bullfrog oil, data that can be explained by the depot effect of the emulsified systems [57]. This phenomenon is responsible for promoting a delay in the in vitro assay due to a slow release of the active compounds incorporated in the emulsion internal phase. Indeed, the surfactant layer in the oil droplets’ surface needs to be unstructured to deliver the internal phase compounds [57,58].

The hypothesis of delay on the in vitro antifungal activity of the emulsified systems was also described in the literature for copaiba essential oil and its nanostructured emulsion. In fact, the pure copaiba essential oil showed MIC values of 0.1083 ± 0.076 mg·mL^−1^ and 0.1083 ± 0.038 mg·mL^−1^ for the *C. glabrata* ATCC 2001 and *C. glabrata* 15V3C, respectively, while the nanoemulsion produced with this oil presented MIC values of 15.6 ± 0.0 mg·mL^−1^ and 0.9736 ± 0.0 mg·mL^−1^ for the respective yeasts [16]. Thus, these results demonstrated that the BBE produced can inhibit the fungal growth of different species of *Candida*, allowing us to suggest that this emulsion can be used as an alternative treatment in OC.

### 3.5. In Vitro Biocompatibility Study

The oral mucosa is an attractive route for drug delivery since it offers a non-invasive way for drug administration and becomes an alternative route when the enteral administration is compromised [59]. In addition, the oral mucosa exhibits a high vascularized area that facilitates the absorption of compounds through the jugular vein that surrounds this site [60], hence bypassing the hepatic first pass metabolism, leading to a possible systemic effect once the active compound is in the bloodstream [35,60,61].

**Figure 2:**
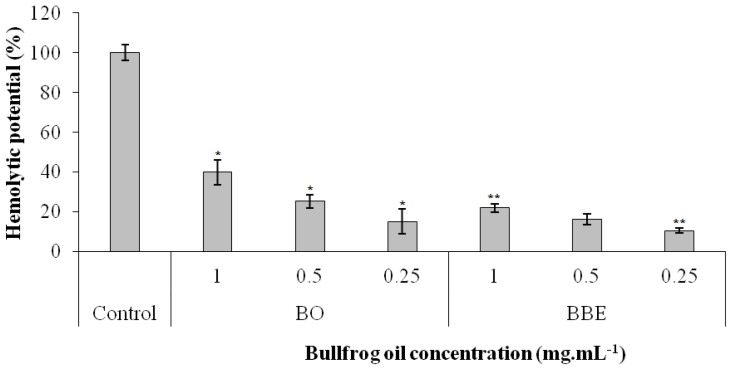
Hemolytic potential of the bullfrog oil (BO), buccal bullfrog oil emulsion (BBE). * Significant statistical difference when compared to the other concentrations in its own group and in comparison to the same concentration of other sample groups (*p* <  0.05). ** Significant statistical difference when compared to the other concentrations in its own group (*p* <  0.05).

Thus, the hemolytic potential of the bullfrog oil and the BBE was evaluated against human erythrocytes to assess the safety and biocompatibility of the produced formulation. Figure 2 shows that the bullfrog oil at the concentration of 1.0 mg·mL^−1^ was able to promote a hemolytic effect of 40 ± 6%, probably due to its rich composition of saturated and unsaturated fatty acids that may interact with the phospholipids in the erythrocytes membranes. This leads to the ability that natural oils have in promoting cell membrane disruption and in increasing the cell membrane permeability, resulting in a complete hemolysis with hemoglobin release [62,63]. On the other hand, as the concentration decreased, the hemolytic effect was reduced significantly. Indeed, at concentrations of 0.5 mg·mL^−1^ and 0.25 mg·mL^−1^, the bullfrog oil hemolytic effect was reduced to 25 ± 3% and 15 ± 6%, respectively, which suggests that the hemolytic effect was concentration dependent, showing the probable biocompatibility of the bullfrog oil at these concentrations. 

Additionally, the BBE was able to reduce the hemolytic effect of the bullfrog oil from 40 ± 6% to 22 ± 2% at 1.0 mg·mL^−1^, highlighting the ability of this system to increase the biocompatibility of the bullfrog oil at this concentration. Indeed, emulsified systems are widely used due to their ability to reduce toxic effects of drugs or natural oils, by promoting a slower release of the active compounds from their internal phase, avoiding an excessive amount of the delivered compound being released at once to promote toxicity or side effects [64,65]. Similar results were reported in the literature in which a submicron emulsion containing bufadienolides was able to reduce the toxicity of this compound [64]. Our group also demonstrated that a bullfrog oil microemulsion loaded with amphotericin B was able to reduce the hemolytic potential of this drug [18]. Furthermore, the BBE did not show significant hemolytic effect at lower concentrations (16 ± 3% and 11 ± 1% for the concentrations of 0.5 and 0.25 mg·mL^−1^, respectively). The Miglyol^®^ 812 and the BME were also tested and did not show significant toxic effect at all concentrations.

Hence, these results show that the developed BBE can reduce the hemolytic effect of the bullfrog oil against erythrocytes, suggesting that this emulsion is biocompatible and may be used on the buccal mucosa even when systemic absorption occurs.

## 4. Conclusions

The results of this work showed that the buccal bullfrog oil emulsion has macroscopic and physicochemical characteristics (droplet size and zeta potential) compatible with stable emulsified systems for over 60 days. In addition, the pharmaceutical excipients used on this formulation provided mucoadhesive properties through its ability of interacting with the mucin, suggesting a buccal mucosa adherence that would allow this system not only to treat local infections, but also to be absorbed and enter the bloodstream. In this regard, the system showed concentration-dependent biocompatibility against human erythrocytes. Furthermore, the produced system inhibited the growth of distinct species of *Candida* responsible for OC.

## Figures and Tables

**Table 1:** Composition of the buccal oil emulsion (BBE and BME).

	Excipients	% (*w*/*w*)	Function
Aqueous phase	Butylhydroxyanisole	0.01	Antioxidant
	Sucralose	0.10	Sweetener
	Tutti-frutti flavoring	0.10	Flavoring
	Sodium benzoate	0.20	Antimicrobial preservative
	Xanthan gum	0.30	Stabilizing agent
	Acesulfame k	0.40	Sweetener
	Tween^®^ 20	3.80	Surfactant
	Propylene glycol	5.00	Humectant
	Distilled water	73.87	Disperser agent
Oily phase	Butylhydroxytoluene (BHT)	0.01	Antioxidant
	Propylparaben	0.02	Antimicrobial preservative
	Span^®^ 80	2.20	Surfactant
	Bullfrog Oil/Miglyol^®^ 812	14.00	Oil

*w*/*w* (weight by weight).

**Table 2:** Mucoadhesive performance of the BBE (buccal bullfrog oil emulsion) and BE (blank emulsion) by the tensile stress test.

Sample	Peak Force	Debonding Distance	*W_ma_*
	mN ± SD	mm ± SD	mN·mm ± SD
BBE	10.15 ± 2.00	1752.36 ± 215.53	1494.04 ± 203.45
BE	7.44 ± 1.53	949.94 ± 92.72	1080.96 ± 204.68

mN (milliNewton); SD (standard deviation); mm (millimeter); Mucoadhesion work (*W_ma_*).

**Table 3:** Droplet size and zeta potential of BBE (buccal bullfrog oil emulsion) and BE (blank emulsion) according to the contact with mucin solutions.

Mucin Concentration (µg·mL^−1^)	Mean Diameter (LD) ± SD (µm)			Zeta Potential ± SD (mV)		
	Mucin Dispersion *	BBE **	BE **	Mucin Dispersion *	BBE **	BE **
0	-	0.320 ± 0.35	0.186 ± 0.02	-	−38.53 ± 6.23	−18.20 ± 1.42
200	0.349 ± 0.291	0.834 ± 0.02	1.138 ± 0.07	−1.79 ± 0.02	−18.10 ± 0.99	−22.75 ± 0.49
250	0.432 ± 0.249	0.944 ± 0.05	1.056 ± 0.23	−2.69 ± 0.05	−19.30 ± 1.41	−15.70 ± 2.26
350	0.779 ± 0.139	1.053 ± 0.02	1.228 ± 0.31	−4.65 ± 0.52	−23.00 ± 4.38	−16.15 ± 2.19

Values were expressed as mean ± Standard Deviation (*n* = 2). LD: laser diffraction; µm (micrometer); mV (millivolts); µg·mL^−1^ (microgram per milliliter); BBE: bullfrog oil emulsion. BE: blank emulsion. * Before contact; ** After contact.

**Table 4:** Minimum inhibitory concentration (MIC) of bullfrog oil and buccal bullfrog oil emulsion.

Yeast	MIC (mg·mL^−1^)	
	Bullfrog Oil	Buccal Bullfrog Oil Emulsion
*Candida albicans* ATCC 90029	0.50	1.00
*Candida dubliniensis* CBS 7987	-	0.50
*Candida glabrata* ATCC 2001	-	1.00
*Candida parapsilosis* ATCC 22019	0.25	1.00
*Candida metapsilosis* ATCC 96143	0.50	0.50
*Candida orthopsilosis* ATCC 96139	0.50	1.00
*Candida tropicalis* ATCC 13803	0.50	1.00
